# Analysis of Retinochoroidal Vasculature in Underweight Women Using Optical Coherence Tomography Angiography

**DOI:** 10.7759/cureus.20562

**Published:** 2021-12-21

**Authors:** Aslı Çetinkaya Yaprak, Lütfiye Yaprak

**Affiliations:** 1 Ophthalmology, Akdeniz University Faculty of Medicine, Antalya, TUR; 2 Ophthalmology, Health Sciences University Antalya Training and Research Hospital, Antalya, TUR

**Keywords:** choroidal capillary, retinal capillary, optical coherence tomography angiography, underweight, choroidal thickness

## Abstract

Introduction

Being underweight (body-mass index < 18.50 kg/m^2^) is associated with significantly higher morbidity than having normal weight. We aimed to investigate the changes in choroid and retinal capillary microcirculation with optical coherence tomography angiography in underweight female subjects compared with an age- and sex-matched population of healthy subjects.

Methods

This prospective, cross-sectional study included 48 eyes of 48 healthy female subjects, of whom 23 were underweight and 25 had normal weight. Optical coherence tomography angiography was used for the evaluation of retinal vessel density, foveal avascular zone, subfoveal central macular thickness, subfoveal choroidal thickness, and choriocapillaris flow area.

Results

The mean choriocapillaris flow area was statistically significantly higher in the underweight group (2.201 ± 0.11 mm^2^) compared to the control group (2.111 ± 0.11 mm^2^) (p=0.005). The subfoveal choroidal thickness was higher in the underweight group (340 ± 61.2 µm) compared to the control group (317 ± 20.8 µm) but the difference was not statistically significant (p=0.099). Similarly, no statistically significant difference was determined between the groups concerning the vessel density, subfoveal central macular thickness, and foveal avascular zone.

Conclusions

Being underweight is associated with a higher subfoveal choroidal thickness and choriocapillaris flow area, independent of additional factors, such as age, axial length, intraocular pressure, and major ocular diseases.

## Introduction

The World Health Organization (WHO) lists and categorizes weight and height related risk groups by their morbidity and mortality risks [1]. As a measure of relative weight, body-mass index (BMI) is easy to obtain. BMI is calculated as weight in kilograms divided by height in meters squared (kg/m2). The current international classification of BMI-related risk groups compiled by WHO includes eight groups: <16.00 kg/m2 (severe underweight), 16.00-16.99 kg/m2 (moderate underweight), 17.00-18.49 kg/m2 (mild underweight), 18.50-24.99 kg/m2 (normal range), 25.00-29.99 kg/m2 (preobese), 30.00-34.99 kg/m2 (obese class I), 35.00-39.99 kg/m2 (obese class II), and ≥ 40.00 kg/m2 (obese class III) [2].

Optical coherence tomography angiography (OCTA) is a relatively new non-invasive and time-efficient imaging technique that provides visualization of perfused vasculature of the choroid and retina [3]. With the use of OCTA software, various measurements, such as vessel density (VD) of the super­ficial and deep capillary plexuses (SCP and DCP), foveal avascular zone (FAZ), outer retina and choriocapillaris flow areas, subfoveal central macular thickness (CMT) and choroidal thickness can be evaluated easily [4, 5].

The relationship between BMI and morbidity has been investigated in previous studies [6]. Obesity is a major public health problem due to its increasing prevalence [7] and its associations with higher morbidity and mortality due to multiple diseases [2,8]. Obesity is also related to various eye diseases, including cataract, age-related maculopathy, diabetic retinopathy, and glaucoma [9]. Previously studies showed that there is an increase in the intraocular pressure (IOP) and a decrease in the anterior chamber depth in obese subjects [10]. In addition to obesity, being underweight is also a health problem. Understanding health consequences related to being underweight has become especially important in some countries where society tends to be slimmer and being underweight is considered crucial [6]. Despite the extensive evaluation of the choroid and retinal capillary microcirculation in obese individuals, there have been relatively few studies performing an OCTA evaluation in underweight patients.

The aim of the present study was to investigate the changes in the choroid and retinal capillary microcirculation with OCTA in underweight female participants compared to a sex- and age-matched population of healthy participants.

## Materials and methods

This prospective, cross-sectional study was obtained the ethics committee of the Antalya Training and Research Hospital, following the tenets of the Declaration of Helsinki and local laws regarding research on human subjects. All participants obtained a signed written informed consent (Clinicaltrials.gov registration ID: NCT04631978).

All subjects underwent a detailed ophthalmologic examination, including the visual acuity, subjective refraction, intraocular pressure (IOP), axial length (AL, Lenstar LS 900, Haag-Streit, Koeniz, Switzerland), anterior and posterior segment examination, and OCTA imaging (Optovue, Freemont, California, USA). To calculate BMI, weight was measured using a certified electronic weighing scale (model number Omron HN-283, Omron, Kyoto, Japan) and a standard anthropometric tape (BioPlus Stature Meter, model number IND/09/2005/815, Bharat Enterprises, Delhi, India) was used to measure height. According to the national guidelines [2], the patients were classified into two underweight groups: Group 1 consisting of those with a BMI of <17.00 kg/m2 and Group 2 comprising those with a BMI of 17.00 to 18.49 kg/m2. The control group was formed with a normal BMI (18.50 to 24.99 kg/m2).

For each participant, the right eye was included in the study. The left eye was selected in case of the right eye met any of the exclusion criteria. The inclusion criteria for the patient group was being underweight (BMI < 18.50 kg/m2). The exclusion criteria were as follows: refractive error higher +3.0 diopters (D) or lower -3.0 D; low image quality; IOP > 21 mm Hg; longer AL (>25 mm); ocular comorbidity; prior ocular surgery; and a comorbid disease that can cause underweight or retinopathy, such as cancer, malnutrition, hypertension, and diabetes mellitus.

Optical coherence tomography angiography

The imaging of all participants was performed with OCTA with a scan beam wavelength of 840 nm, scan speed of 70,000 A/s, and bandwidth of 45 nm. All measurements were performed between 09:00 - 11:00 to minimize intraday differences.

To evaluate the microvascular structures, ‘HD Angio Retina’ protocol was used centered 6×6 mm grids onto the fovea-centered macular region. The images of low quality (signal strength index < 6/10) with either incorrect segmentation or significant motion artifact were excluded. The full-thickness retinochoroidal scans automati­cally were segmented into the super­ficial retinal plexuses, deep retinal plexuses, choriocapillaris (CC) and outer retina. The vascular densities in the superficial retinal vascular zones and deep retinal vascular zones, foveal density (FD), and FAZ were calculated automatically. The two independent retinal specialists (A.Ç.Y., L.Y.) calculated the choroidal thickness manually from the outer edge of the hyperreflective retinal pigment epithelium (RPE) to the choroid-scleral junction, and the average value was recorded.

Statistical analysis

The statistical analysis was performed using SPSS, version 21.0 (IBM Corp, Armonk, USA). To define the sample, continuous variables were expressed as mean ± standard deviation, median (minimum-maximum) and categorical variables as number and percentage. The normality assumption for the independent variables was checked with the Shapiro-Wilk and Bonferroni tests. In the comparison of the continuous data, the ANOVA and independent-samples t-tests were applied to the normally distributed data and the Kruskal-Wallis and Mann-Whitney U tests were used for the data with non-normal distribution. Data analyses were evaluated at the 95% confidence interval, and considering a p-value of < 0.05 as statistically significant.

## Results

A total of 48 eyes of 48 healthy female subjects were included in this study. Twenty-three (47.9%) individuals were in the underweight group and 25 (52.1%) were in the control group. In the underweight group, 10 had a BMI of <17.00 kg/m2 (Group 1) and 13 17.00-18.49 kg/m2 (Group 2). The mean age was 26.8 ± 6.9 years in the underweight group and 24.4 ± 6.2 years in the control group (p = 0.96). No statistically significant difference was determined between the groups in respect of age, spherical equivalent, IOP, central corneal thickness (CCT), and AL (Table 1). 

**Table 1:** Demographic and ocular parameters of the underweight and control groups Normally distributed values are presented as means ± standard deviation and non-normally distributed values are presented as median (range) IOP intraocular pressure, CCT central corneal thickness, BMI body mass index *Independent-samples t-test; **Mann-Whitney U test

Parameters	Underweight (n = 23)	Control (n = 25)	p
Age (years)	26.8 ± 6.9	24.4 ± 6.2	0.96*
IOP (mmHg)	14.9 (range, 10.7-20)	15 (range, 12-20)	0.771**
CCT (µm)	532.3 ± 34.3	543.8 ± 28.9	0.215*
Axial length (mm)	23.33 ± 1.02	23.41 ± 0.81	0.779*
Spherical equivalent (dpt)	− 0.32 ± 0.7	− 0.34 ± 0.6	0.813*
BMI (kg/m^2^)	17.4 (range, 14.2-18.4)	23.6 (range, 22.3-24.9)	<0.001**

**Figure 1:**
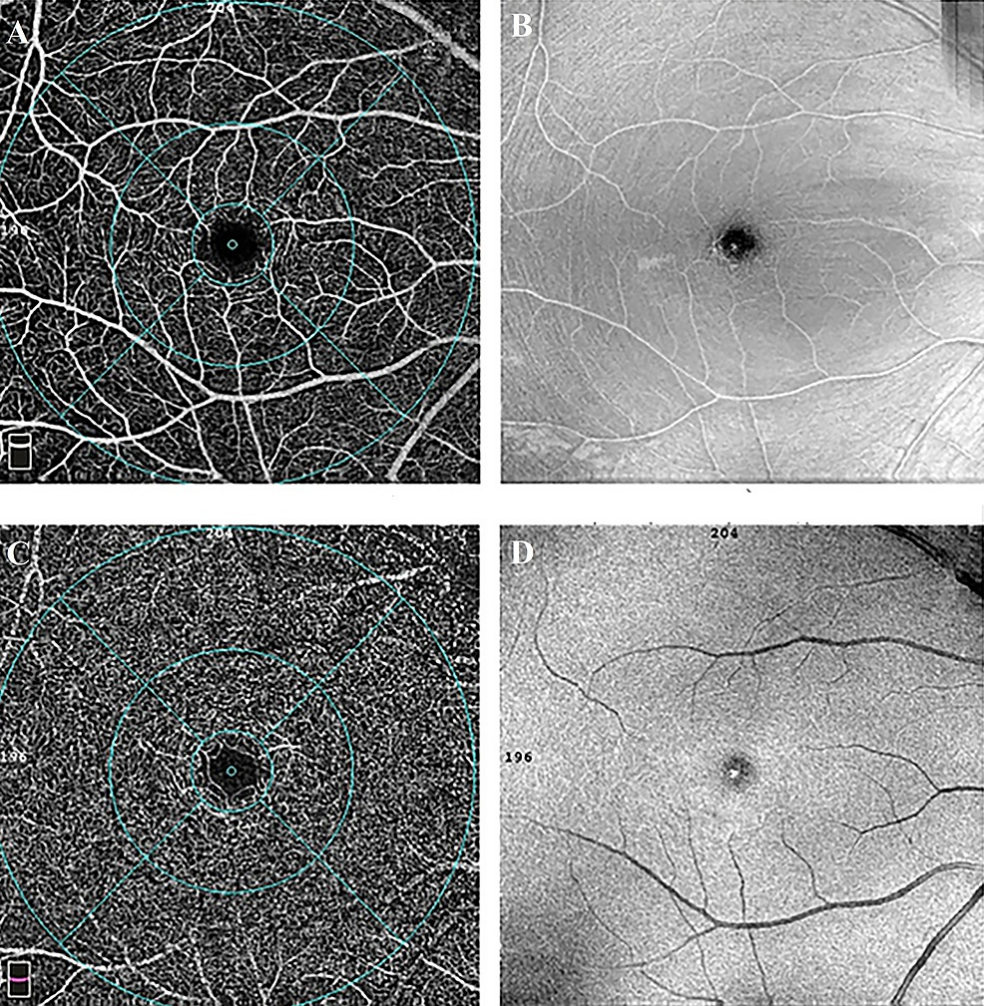
Maps of superficial and deep vessel density and retinal thickness in an underweight female subject. A, B: Superficial capillary plexus; C, D: Deep capillary plexus Using the density measure feature, AngioVue software (OptoVue, Fremont, USA) can provide an automatically detailed map which calculates the percentage areas occupied by superficial and deep vessels in the whole macular area, fovea, parafovea, and perifovea.

**Figure 2:**
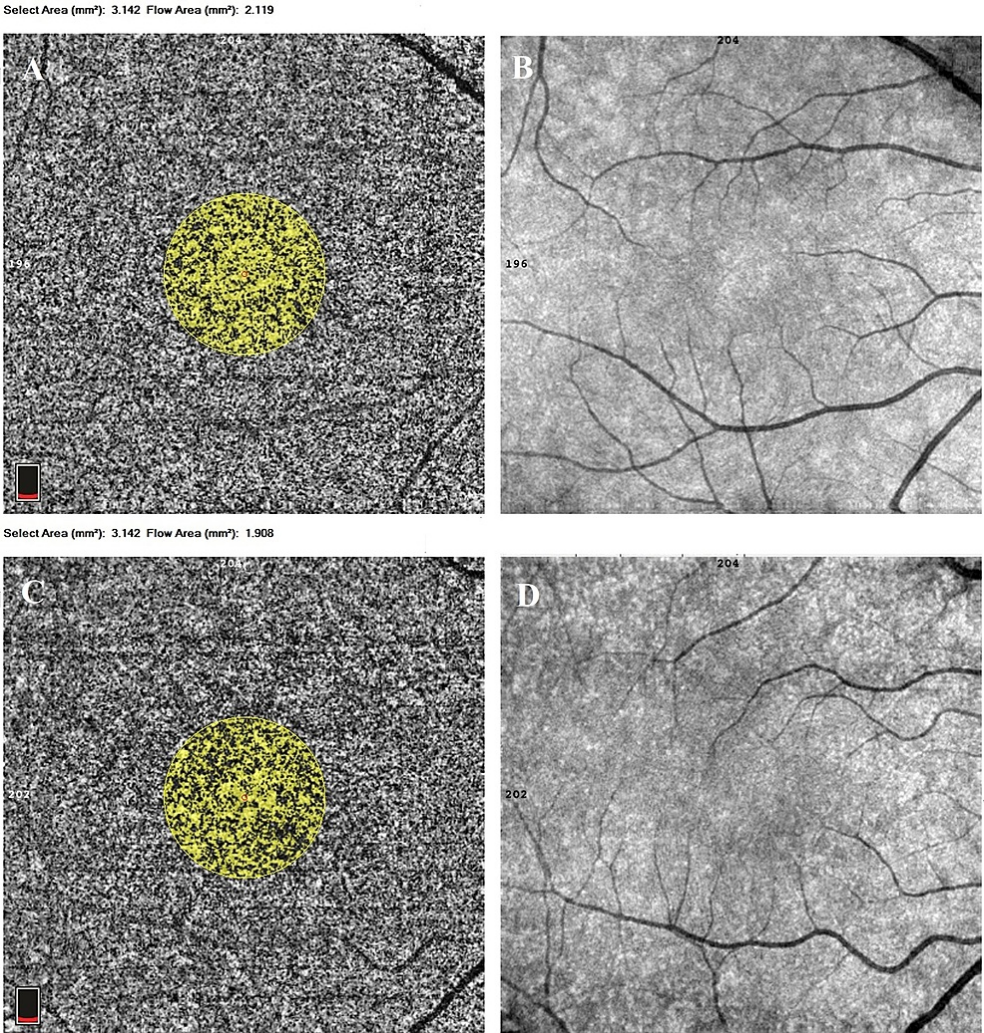
Flow measurements in the choriocapillaris A, B: Flow measurements in the choriocapillaris of an underweight female subject; C, D: Flow measurements in the choriocapillaris of a healthy subject The choriocapillaris flow area was automatically delimitated by the software

**Figure 3:**
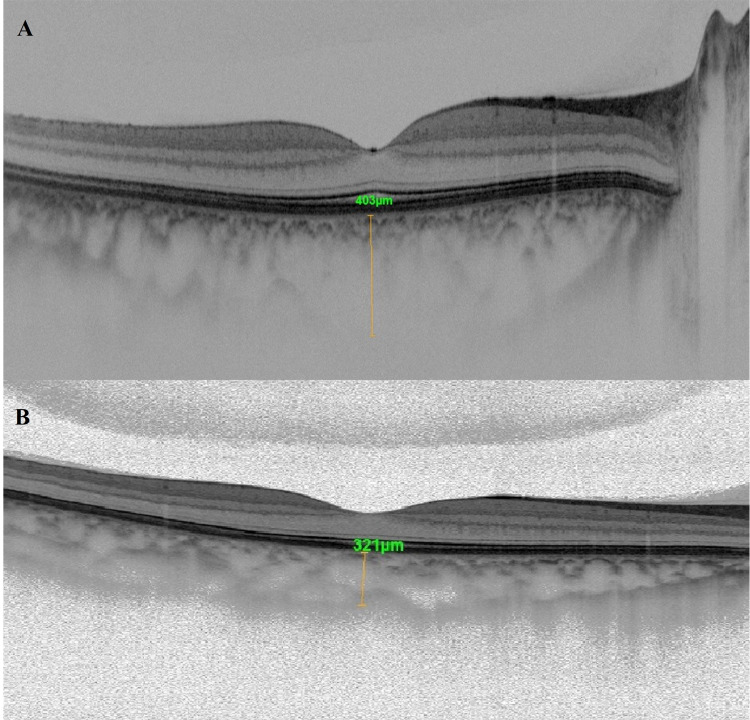
Choroidal thickness A. Choroidal thickness of an underweight female subject; B. Choroidal thickness of a healthy subject.

The whole, foveal, parafoveal and perifoveal VDs of the SCP and DCP measurements and the CMT of the participants are shown in Table 2 and Fig. 1. No statistically significant difference was found between the groups in terms of VD. The mean FAZ measurements were 0.3 ± 0.11 mm2 in the underweight group and 0.33 ± 0.11 mm2 in the control group. The difference between the groups was not statistically significant (p = 0.342). The mean CC flow area was statistically significantly higher in the underweight group (2.201 ± 0.11 mm2) compared to the control group (2.111 ± 0.11 mm2) (p = 0.005) (Fig. 2). The subfoveal choroidal thickness was higher in the underweight group (340 ± 61.2 µm) compared to the control group (317 ± 20.8 µm), but not at a statistically significant level (p = 0.099) (Table 2 and Fig. 3). 

**Table 2:** Comparison of the optical coherence tomography angiography measurements between the underweight and control groups Normally distributed values are presented as means ± standard deviation and non-normally distributed values are presented as median (range) VD vessel density, SCP super­ficial capillary plexus, DCP deep capillary plexus, CMT central macular thickness, FAZ foveal avascular zone *Independent-samples t test; **Mann–Whitney U test

Parameters	Underweight (n = 23)	Control (n = 25)	p
SCP VD-whole (%)	51.3 ± 1.8	50.9 ± 2.5	0.622*
SCP VD-fovea (%)	18.67 ± 6.51	18.02 ± 5.81	0.713*
SCP VD-parafovea (%)	53.9 (range, 47.5-57.5)	53.8 (range, 43-56,9)	0.599**
DCP VD-whole (%)	56.2 (range, 47.8-64.2)	55 (range, 40.2-59.8)	0.765**
DCP VD-fovea (%)	36.3 ± 7.9	34.8 ± 7.6	0.505*
DCP VD-parafovea (%)	58 ± 3.1	57.6 ± 3.7	0.643*
CMT (mm)	251.3 ± 24.9	247.1 ± 18.2	0.507*
FAZ (mm^2^)	0.30 ± 0.11	0.33 ± 0.11	0.342*
Choriocapillaris flow area (mm)	2.201 ± 0.11	2.111 ± 0.11	0.005*
Subfoveal choroidal thickness (µm)	340 ± 61.2	317 ± 20.8	0.099*

The mean FAZ, subfoveal choroidal thickness and CC flow area measurements of the control and underweight groups are shown in Table 3. The mean CC flow area thickness was higher in Group 1 than in Group 2 and in Group 2 than in controls (p = 0.022). The subfoveal choroidal thickness was higher in Group 1 than in Group 2 and in Group 2 than in controls, but the differences were not statistically significant (p = 0.187). 

**Table 3:** Comparison of the optical coherence tomography angiography measurements between the two underweight groups and the controls Values are presented as means ± standard deviation FAZ foveal avascular zone *ANOVA test

Parameters	Group 1 (n = 10)	Group 2 (n = 13)	Control (n = 25)	p
FAZ (mm^2^)	0.298 ± 0.12	0.306 ± 0.95	0.332 ± 0.11	0.631*
Choriocapillaris flow area (mm)	2.204 ± 0.11	2.199 ± 0.10	2.111 ± 0.11	0.187*
Subfoveal choroidal thickness (µm)	347.4 ± 63.4	335.4 ± 61.6	317.7 ± 20.8	0.022*

## Discussion

In the current study, we found that the subfoveal choroidal thickness measured by OCTA was higher among the underweight women (BMI < 18.50 kg/m2) compared with those with normal weight (BMI: 18.50-24.99 kg/m2). Similarly, the CC flow area was statistically significantly higher in the underweight group.

Anthropometry is the single most portable, universally applicable, inexpensive, and non-invasive method available to assess the proportions, size and composition of the human body [11]. It reflects both health and nutrition and can assist in the prediction of predict performance, health, and survival [11]. WHO has suggested that the classifications of bodyweight should contain levels of underweight and grading of overweight that are related to increased risk of some non-infectious diseases [1,8]. These classifications are mostly based on BMI, as the most widely adopted metric for defining underweight and overweight.

The choroid is primarily a vascular structure consisting of an outer macrovascular layer and an inner capillary layer called the CC, which lies immediately below Bruch’s membrane and extends from the ora serrata to the optic nerve. The function ascribed to the choroid is to supply oxygen and nourishment to the outer retina. Choroidal defects cause degenerative changes and neovascularization. Choroidal thickness has been affected by some variables, such as age, sex, refraction, and AL, as well as some local and/or systemic diseases [12-15]. Previous studies in obese participants have shown that the subfoveal choroidal thickness is wider in patients with obesity compared to the healthy control subjects [16,17]. In another study displayed that there was no significant difference between underweight patients (BMI <18.50 mm/kg2) compared to the patients with normal weight (BMI: 18.50-24.99 mm/kg2) in the subfoveal choroidal thickness [18].

Normative databases for the subfoveal choroidal thickness have also been presented in the literature. The mean subfoveal choroidal thickness previously was reported 312.9 ± 65.3 mm, 265.5 ± 82.4 mm, and 348 ± 68 mm [18-20]. In our study, this thickness was found to be 340 ± 61.2 mm in the underweight group.

The results of our study demonstrated that the underweight patients had a higher choroidal thickness (Table 2). However, this was not significantly different from the control group. Previous studies showed that the choroidal thickness was affected by weight loss in obese patients. The mean choroidal thickness significantly increased in patients who underwent bariatric surgery while there was no statistically significant difference in those that received conservative treatment. The thickening of the choroid can be attributed to the characteristic features of autoregulation in choroidal flow and altered hemodynamics [21]. Similarly, another study was speculated that the changes in the choroid thickness could be the result of altered choroidal blood flow, vascular permeability, relaxation and contraction of the choroidal stromal muscle cells, and in the production of osmotically active particles [22].

Nitric oxide (NO), which is a key endothelium-derived vasodilator, increases tissue perfusion when there is a need for metabolic demand [23]. Sympathetic activation and noradrenaline release scale the choroidal blood flow down, NO signaling and the subsequent parasympathetic efferent nerve activation, on the other hand, causes a scale-up [24]. Obesity diminishes NO activity and results in impaired vasodilatation [25]. In our study, the CC flow area was statistically significantly higher in the underweight group. This may have been caused by the level of NO in this group.

We initially compared the retinal vascular parameters in underweight patients to a control group with normal weight. Our results demonstrated that the underweight patients had higher vascular retinal parameters, such as the VDs of SCP and DCP (Table 2). However, there was no significant difference between the underweight and control groups in terms of these parameters.

The strengths of this study include underweight women having no ocular and systemic disease, thus being free of confounding factors. We also included age- and sex-matched controls with normal weight, and there was no statistically significant difference about spherical equivalent, IOP, CCT, and AL between the groups. However, our study also has certain limitations, such as a relatively small sample size and cross-sectional design, pathogenesis of being underweight including several unknown genetic and hormonal factors, and the choroid being a vascular tissue that may be affected by systemic and/or local factors. Since choroidal tissue may differ between males and females, we evaluated only women in our study. Additionally, it can be suggested that underweight men have changes in choroid; however, further studies to be conducted with men are warranted to confirm this idea.

## Conclusions

In conclusion, using OCTA measurements, our study demonstrated that being underweight was associated with a higher subfoveal choroidal thickness and CC flow area, independent of additional factors, such as age, axial length, IOP, and major ocular diseases. However, it remains unclear how this information can be applied to participants and benefit underweight management. Our results suggest that the choroidal thickness and CC flow area measurements play a predictive role, and BMI should be included among the parameters that may affect results in underweight women. A prospective, large sample size follow-up study is necessary to test our hypothesis and to validate the results of the current clinical trial.
